# Genetic and In Vitro Characteristics of a Porcine Circovirus Type 3 Isolate from Northeast China

**DOI:** 10.3390/vetsci10080517

**Published:** 2023-08-10

**Authors:** Menghang Wang, Ying Yu, Jianan Wu, Shujie Wang, Luis G Giménez-Lirola, Pablo Piñeyro, Yu Wang, Hongliang Cui, Xijun He, Jeffrey J. Zimmerman, Yabin Tu, Xuehui Cai, Gang Wang

**Affiliations:** 1 Heilongjiang Research Center for Veterinary Biopharmaceutical Technology, State Key Laboratory for Animal Disease Control and Prevention, Harbin Veterinary Research Institute, Chinese Academy of Agricultural Sciences, Harbin 150069, China; wmhtcs@163.com (M.W.);; 2 College of Veterinary Medicine, Qingdao Agricultural University, Qingdao 266109, China; 3 Department of Veterinary Diagnostic and Production Animal Medicine, College of Veterinary Medicine, Iowa State University, Ames, IA 50011, USA; 4 Shandong Provincial Key Laboratory of Animal Biotechnology and Disease Control and Prevention, College of Veterinary Medicine, Shandong Agricultural University, Tai’an 271002, China

**Keywords:** porcine circovirus, PCV3 isolation, PCV-like particles, genomic characteristics

## Abstract

**Simple Summary:**

Although several studies of porcine circovirus 3 (PCV3) have been conducted, there is little information regarding the pathogenicity of PCV3, in large part because of the small number of PCV3 isolates available. In this study, a PCV3 strain named PCV3-DB-1, belonging to the PCV3a branch, was isolated from PK-15 cells and with 24 alanine and 27 lysine in the Cap protein of this strain, which is unique. PCV-like particles were subsequently observed using electron microscopy and the RNA replication of PCV3 was confirmed in PK-15 cells using situ hybridization RNA scope. This is the first report on the isolation and in vitro characteristics of PCV3-DB-1 with 24A and 27K of the Cap protein. PCV3 was reported in 2015 on a sow farm experiencing PDNS-like and reproductive failure clinical symptoms in the United States, and was subsequently found in most swine-producing countries in Europe, Asia, and America. Although neurological disorders, as well as respiratory and enteric disease, have been described in association with PCV3 infection, multisystemic inflammation and reproductive failure are the most concerning clinical presentations. Studies reproducing and characterizing PCV3 clinical disease and pathogenesis under experimental conditions are scarce, in large part because of the difficulty of isolating PCV3. In this study, we isolated PCV3-DB-1 in PK-15 cells and characterized them in vitro. By using electron microscopy, PCV-like particles were discovered, and in situ *hybridization* RNA sequencing showed PCV3 replication in PK-15 cell culture. Based on phylogenetic analysis of PCV3 isolates from the Heilongjiang province of China, PCV3-DB-1 with 24 alanine and 27 lysine in the Cap protein was originally isolated and determined to belong to the clade PCV3a.

**Abstract:**

Porcine circovirus 3 (PCV3) is an emerging virus first discovered in the United States in 2015, and since then, PCV3 has been found in many regions of the world, including America, Asia, and Europe. Although several PCV3 investigations have been carried out, there is a lack of knowledge regarding the pathogenicity of PCV3, mostly due to the limited number of PCV3 isolates that are readily available. In this study, PCV3-DB-1 was isolated in PK-15 cells and characterized in vitro. Electron microscopy revealed the presence of PCV-like particles, and in situ *hybridization* RNA analysis demonstrated the replication of PCV3 in PK-15 cell culture. Based on phylogenetic analysis of PCV3 isolates from the Heilongjiang province of China, PCV3-DB-1 with 24 alanine and 27 lysine in the Cap protein was originally isolated and determined to belong to the clade PCV3a.

## 1. Introduction

Circoviruses (CV), the smallest known autonomously replicating viruses, belong to the genus *Circovirus* in the *Circoviridae* family [1]. Circoviruses encode three to five open reading frames on opposite strands of a double-stranded DNA replicative intermediate [2,3]. Four porcine circoviruses (PCV) have been recognized to date: PCV1, PCV2, PCV3, and PCV4 [1,4,5,6,7]. PCV1 is not pathogenic in swine but is a common contaminant of the porcine kidney cell line (PK-15) [8]. PCV2 infection causes a range of clinical diseases, including post-weaning multisystemic wasting syndrome (PMWS), porcine dermatitis and nephropathy syndrome (PDNS), and reproductive failure [9]. PCV3 was reported in 2015 on a sow farm experiencing PDNS-like and reproductive failure clinical signs in the United States [5] and was subsequently found in most swine-producing countries in Europe, Asia, and America [10,11,12]. Both PCV2 and PCV3 were detected with high frequency in different tissues of wild boar [13,14], confirming that PCV2 and PCV3 may pose a persistent risk to the swine industry. PCV4 was detected in 2019 in China in association with clinical respiratory and enteric disease and PDNS [7].

More than ten provinces in China have reported the presence of PCV3 [15,16,17,18]. Subclinical PCV3 infection is characterized by viremia or detection of PCV3 in multiple tissues without evident clinical signs or lesions in pigs of all ages [19]. Although neurological disorders, as well as respiratory and enteric disease, have been described in association with PCV3 infection, multisystemic inflammation and reproductive failure are the most concerning clinical presentations. Studies reproducing and characterizing PCV3 clinical disease and pathogenesis under experimental conditions are scarce [20,21], in large part because of the difficulty of isolating PCV3. Hence, two studies have reported successful isolation of PCV3 [20,22], and only one provides further characterization of an isolate in vivo [20].

Significant advances associated with understanding the virus’s genetic structure and evolution have occurred since its first description. A consistent mutation in amino acids of the cap protein has been found through phylogenetic analyses of both recent and historical samples, which suggests that mutations in the amino acids at positions 24 and 27 of the cap protein could serve as molecular markers to divide PCV3 into the three clades PCV3a, PCV3b, and PCV3c [23]. In addition, PCV3a has been further subdivided into three subclades (PCV3a1, PCV3a2, and PCV3a3) based on evolutionary relationships and other molecular characteristics of the cap protein [23]. However, most of these reports were based on molecular detection and not viruses isolated from clinical cases. Thus, the association between the proposed clades and their phenotypic characteristics is incomplete in terms of pathogenicity. This report describes the genetic and in vitro characterization of the first reported PCV3 isolate from the Chinese province of Heilongjiang.

## 2. Materials and Methods

### 2.1. Virus Isolation

PCV3 (PCV3-China/DB-1/2017, MH286898) was isolated retrospectively from the lungs of two 10-day-old neonatal piglets in a commercial pig herd in the northeast Chinese province of Heilongjiang. Reported clinical signs were characterized by dyspnea, anorexia, lethargy, and an increment in >50% mortality. The presence of PCV2 [24], PRRSV [25], CSFV [26], PPV [27], swine influenza virus (AIV) [28], and *Mycoplasma hyopneumoniae* [29] infection was ruled out based on specific PCR detection. Specifically, the isolate was obtained via a single passage in a PCV1- and PCV2-free porcine kidney cell line (PK-15, ATCC^®^ CCL-33™, Hong Kong, China). Lung tissue was processed as previously described [30]. Briefly, PK-15 cells were cultured in a 25 cm^2^ flask containing DMEM with 10% fetal bovine serum (Gibco, Invitrogen Corporation, Waltham, MA, USA), 100 U of penicillin/mL, and 100 μg streptomycin/mL at 37 °C and 5% CO_2_. When PK-15 cells reached ~40% confluency, the medium was removed, and the cells were washed three times with sterile phosphate-buffered saline (PBS, pH 7.4). Next, 2 mL of filtered supernatant was added and the sample was incubated at 37 °C and 5% CO_2_ for 2 h. The inoculum was replaced with a fresh medium with 2% fetal bovine serum, and the cells incubated for 72–96 h at 37 °C and 5% CO_2_. The virus was recovered through three freeze–thaw cycles and passaged three times in PK-15 cells. The cell lysate was confirmed to be free of PCV2 [24], PCV1 [31], classical swine fever virus [26], porcine parvovirus [27], and pseudorabies virus [32] using real-time PCR.

### 2.2. Evaluation of Viral Particles in PK-15 Cells Using Electron Microscopy

PK-15 cells were inoculated with ~1.18 × 10^5^ genomic copies/mL of PCV3 DB-1, mock-infected with cell culture lysate in DMEM containing 2% fetal bovine serum (Gibco, Invitrogen Corporation), and incubated for 72–96 h at 37 °C and 5% CO_2_. Samples for electron microscopy studies were prepared according to the Veterinary Research Institute’s (Harbin, China) standard operating procedures. Briefly, the infected and mock-infected PK-15 cells were harvested and fixed with 2.5% (*w*/*v*) glutaraldehyde at 4 °C for 2 h and subsequently fixed with 1% OsO4. After being dehydrated in an acetone gradient, the samples were infiltrated using eponate resin at room temperature and embedded at 70 °C overnight for polymerization in beem capsules. Ultrathin sections 70 nm thick were cut with an Ultracut Eultramicrotome (Leica, Wetzlar, Germany) and stained with 2% uranyl acetate for 15 min and lead citrate for 10 min. After drying overnight, the samples were evaluated using conventional transmission electron microscopy (TEM, H7650, HITACHI, Tokyo, Japan).

### 2.3. Viral Characterization In Vitro through In Situ Hybridization (RNAscope) in Cell Culture

The PCV3 DB-1 isolate was passaged three times in PK-15 cells under the same conditions described above for virus isolation. After inoculation, the cells were incubated at 37 °C and 5% CO_2_ for 72–96 h and then fixed with 10% Neutral Buffered Formalin (NBF). According to the manufacturer’s instructions, viral replication was confirmed using a kit from Advanced Cell Diagnostics, Inc., (Hayward, CA, USA), RNAscope^®^ 2.5 HD. The RNAscope^®^ probes (catalog no. 463961 or 530431) targeting PCV3 RNA, along with the control probes, were designed and synthesized by Advanced Cell Diagnostics. Visualization of the samples was carried out with a microscope from Olympus Corporation, Tokyo, Japan.

### 2.4. Genome Sequence Analysis

Four pairs of primers were synthesized for complete genome amplification through PCR using methods previously reported [5]. Genotype identification of PCV3 was performed using phylogenetic tree deduction based on complete coding sequences (ORF1 + ORF2) [33]. To perform phylogenetic analysis, 58 PCV3 coding sequences originating from Sus scrofa (from NCBI) were downloaded. Alignment was carried out using default parameters in the Clustal W program (Lynnon Co., Zhuhai, China, DNAMAN 6.0 software). A phylogenic tree was constructed through the maximum likelihood method with 1000 bootstrap replicates in the MEGA program (v5.0) [34].

## 3. Results

**Figure 1:**
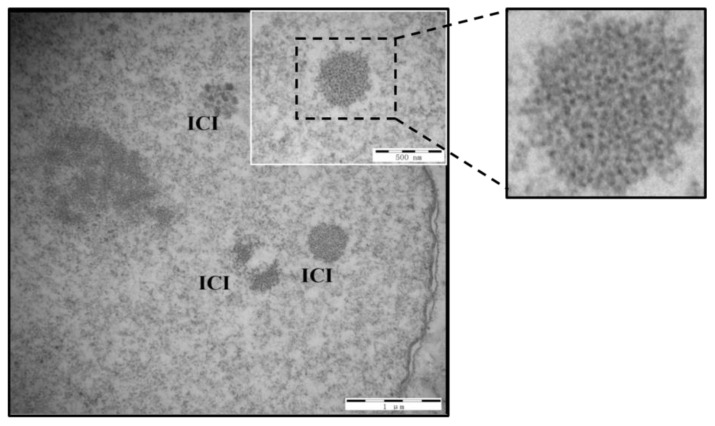
Electron micrographs of PCV3-infected PK-15 cells at 72 h post-inoculation. Viral-like particles are grouped in paracrystalline arrays within a large, complex intracytoplasmic inclusion (ICI) body (insert at higher magnification). Scale bar: 1 μm.

**Figure 2:**
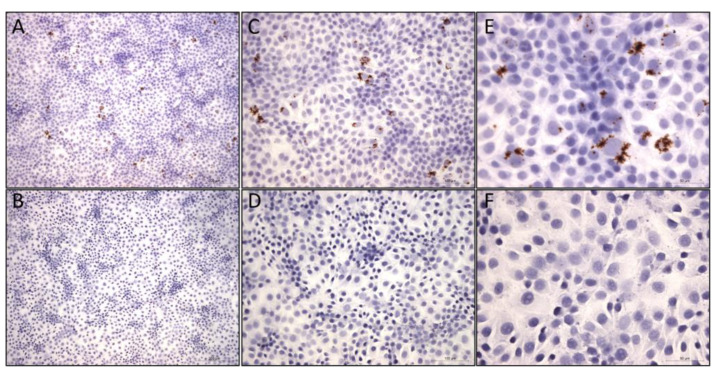
Isolation and identification of PCV3 DB-1 in PK-15-infected cells. Brown positive signals were found in the cytoplasm of PK-15-infected cells of PCV3 DB-1 isolates via ISH RNA scope at 10× magnification (**A**), 20× magnification (**C**), and 40× magnification (**E**). No positive signals were detected in the mock-inoculated PK-15 cells (**B**,**D**,**F**).

The present study describes the retrospective isolation of PCV3 (PCV3 isolate DB-1) from clinically affected animals and its replication in PK-15 cells. Specifically, the PCV3 isolate DB-1 was cultured three times in PK-15 cells, and productive virus replication was confirmed using electron microscopy and ISH RNAscope^®^ on virus obtained from each passage. The evaluation of PK-15 cells infected with PCV3 DB-1 using electron microscopy demonstrated the presence of PCV-like particles after a 72–96 h incubation at 37 °C and 5% CO_2_. Organized intracytoplasmic inclusion bodies (ICIs) 0.2–0.5 μm in diameter, forming large and complex electron-dense bodies, contained recognizable VLPs (Figure 1). No VLPs were observed in mock-infected cells. Likewise, virus replication in PK-15 cells was confirmed through ISH RNA scope on each passage. At 72–96 h of incubation, ISH revealed intracytoplasmic hybridization signals consistent with genome replication of PCV3 in vitro (Figure 2A,C,E).

**Figure 3:**
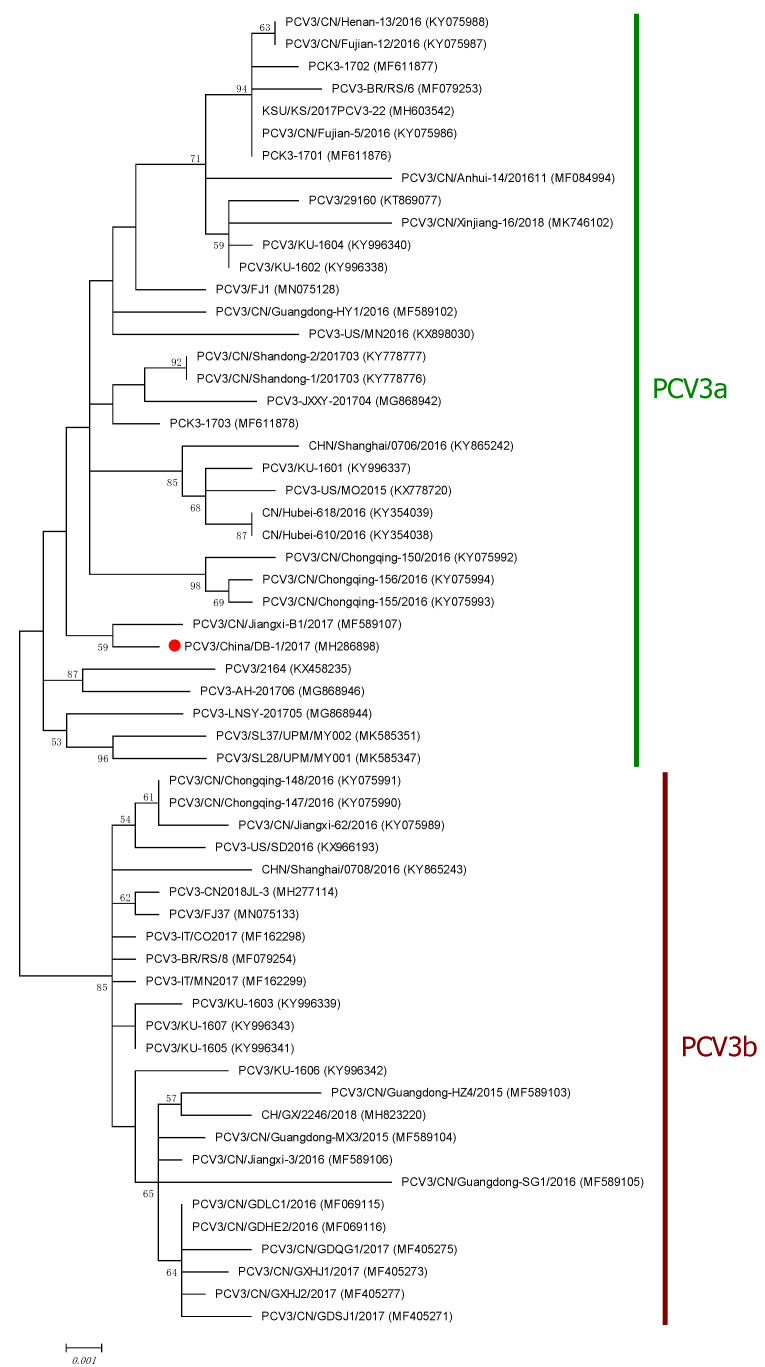
Phylogenetic analysis based on the complete coding sequences of the PCV3 isolates. MEGA 5.0 was used to reconstruct the ML tree using 1000 bootstrap trials. Bootstrap values are represented by the values along the branches.

Phylogenetic characterization showed that PCV3 DB-1 is a member of the PCV3a clade, but it is the only known PCV3 isolation of the amino acids 24 alanine (A) and 27 lysine (K) of the Cap protein in the NCBI database. Specifically, the nucleotide similarity between PCV3 DB-1 and the PCV3 sequences deposited in GenBank^®^ (containing 58 sample sequences) was 97.3–99.7% (Figure 3). PCV3 DB-1 showed the highest (99.7%) and lowest (97.3%) nucleotide identities with PCV3/CN/Jiangxi-B1/2017 (MF589107) and PCV3/CN/Guangdong-SG1/2016 (MF589105), respectively.

## 4. Discussion

ICIs, structures that can be observed within the cytoplasm of infected cells, are often associated with viral infection and are the result of the interaction between the virus and host cell. In this study, PCV3-infected cells showed the presence of virus particles with the same size and morphology as porcine circovirus [8], forming large and complex electron-dense bodies. Similarly, PCV3-induced inclusion bodies appear as round or irregular structures within the cytoplasm of infected primary porcine kidney cells [22]. ICI observation in histopathological samples or cell cultures can support the identification and confirmation of PCV3’s presence. The exact composition of the inclusion bodies in PCV3-infected cells has not been fully characterized yet. The understanding of PCV3 and its intracytoplasmic inclusion bodies is still evolving, and further research is needed to fully characterize their composition, formation process, and diagnostic significance.

ISH RNAscope is a molecular technique used to detect and visualize specific RNA molecules within cells or tissue sections, providing insights into the cellular distribution and expression patterns of the target RNA. We confirmed the PCV3 replication in the PK-15 cells using ISH RNAscope and revealed intracytoplasmic hybridization signals consistent with the genome replication of PCV3 in vitro. Similarly, the signals generated by the PCV3 RNA probes indicate the presence and localization of PCV3 RNA within the primary porcine kidney cells [22]. ISH RNA scope allows researchers to directly visualize PCV3 RNA within the cellular context, providing valuable information about viral replication sites, tissue tropism, and cellular response to infection. It is a powerful tool for studying the pathogenesis and molecular biology characteristics of PCV3.

Phylogenetic analysis can identify distinct genotypes, lineages, or subgroups in the classification and clustering of PCV3 strains based on their genetic relatedness, which provides insights into the geographic distribution and evolutionary dynamics of PCV3. The genetic diversity of PCV3 seems to increase as the number of sequences reported from different countries does too; therefore, the current phylogenetic classification remains dynamic and subject to frequent changes. Nevertheless, PCV3 is a recently identified virus, but it has been found retrospectively since 1966 in China [23], 1967 in Brazil [35], 1996 in Spain [36], and 2006 in Thailand [37]. The cap protein of PCV3 exhibits genetic variation, with different strains or variants of the virus having slight differences in their cap protein sequences. These genetic variations may have implications for viral pathogenicity and antigenicity. Studying the cap protein of PCV3 is essential for understanding its structural properties, antigenicity, and potential role in virus–host interactions. Importantly, besides the phylogenetic differences observed in the field, there are no studies that support phenotypic differences amongst different clades. PCV3a has been associated with cases of reproductive failure [38,39], PDNS, and PCVAD [40]. Thus, further research is required to determine if members of the PCV3a clade with 24 alanine and 27 lysine within the Cap might have distinctive phenotypic characteristics that can be related to certain clinical manifestations.

In our study, immortalized cell lines (PK-15, Vero, ST, and DF1) and primary cells (PAM, PBMC, and bronchial epithelial cells) were used to isolate and passage the virus (Tables S1 and S2). The qPCR assay results showed that the PK-15 cell line was more suitable for the proliferation of PCV3-DB-1 isolation for a limited number of passages [41]. The screening of susceptible single-cell clones from the PK-15 cell line may be necessary for in vitro isolation of PCV3. PCV3 has circulated in the global swine population for several decades or more and will undoubtedly continue to do so in the future, bringing with it the potential for future clinical outbreaks. There is a broad spectrum of clinical signs associated with PCV3 infection, ranging from inapparent or subclinical infection to reproductive failure and PDNS-like signs in sows [5] and multisystemic and cardiac inflammation in grower–finisher pigs [42,43]. Interestingly, the experimental inoculation of CDCD piglets did not result in significant clinical disease and produced only mild histological changes associated with myocarditis and systemic perivasculitis [18,19]. It is known that the severity of the clinical disease induced by PCV2 infection is related, amongst other factors, to co-infections with other swine pathogens, including PRRSV, IAV, and PPV [44]. Whether PCV3-associated disease is related to the interactions and synergy of other infectious factors (co-infections), environmental factors related to production systems and husbandry, and pig-related factors like genetics, age, and immune status warrants further investigation.

## 5. Conclusions

In conclusion, this report describes the retrospective isolation of PCV3 in pigs with respiratory distress and its characterization in vitro. A phylogenic analysis demonstrated that PCV3a isolation with 24 alanine and 27 lysine of the Cap protein in this case is the first reported in the NCBI database of PCV3 so far. Although numerous studies have reported PCV3 in swine production systems worldwide, a complete understanding of the pathogenesis of PCV3 and the risk factors that trigger different clinical presentations in the field requires further investigation.

## Data Availability

Not applicable.

## Supplementary Materials

The following supporting information can be downloaded at: https://www.mdpi.com/article/10.3390/vetsci10080517/s1, Table S1: Detection of PCV3-DB-1 passage in cell lines by qPCR; Table S2: Detection of PCV3-DB-1 passage in primary cells by qPCR.
