# Supplementary material for: Genetic and In Vitro Characteristics of a Porcine Circovirus Type 3 Isolate from Northeast China
Source: Vet Sci. 2023 Aug 10;10(8):517. doi: 10.3390/vetsci10080517 (PMC10459391; doi:10.3390/vetsci10080517)
Supplement: Supplementary file 1 [file vetsci-10-00517-s001.zip › vetsci-2412439-supplementary.pdf]

veterinary sciences

Supplementary Materials

Table S1 Detection of PCV3-DB-1 passage in cell lines by qPCR.

Cell lines	PCV3 qPCR Results						
	P1	P2	P3	P4	P5	P6	Control
PK-15	14.5	20.3	22.4	23.6	27.4	32.1	—
Vero	33.3	—	—	—	—	—	—
ST	18.4	34.7	—	—	—	—	—
DF1	—	—	—	—	—	—	—

—: higher than Ct cut-off value; Each number represents the average Ct values generated from at least three times detection. PK-15: porcine kidney cell line; Vero: African green monkey kidney cell line; ST: Swine testicular cell line; DF1: chicken embryonic fibroblast cell line.

Table S2 Detection of PCV3-DB-1 passage in primary cells by qPCR.

Primary Cells	PCV3 qPCR Results						
	P1	P2	P3	P4	P5	P6	Control
PAM	27.3	30.5	35.2	—	—	—	—
PBMC	—	—	—	—	—	—	—
BEpC	25.8	34.7	—	—	—	—	—

—: higher than Ct cut-off value; Each number represents the average Ct values generated from at least three times detection. PAM: pulmonary alveolar macrophages; PBMC: peripheral blood mononuclear cell; BEpC: epithelial cells bronchial epithelial cells.
